# Bis[tris­(pyridin-2-yl)amine]­iron(II) tris­(di­cyano­methyl­idene)methane­diide

**DOI:** 10.1107/S241431462001278X

**Published:** 2020-09-25

**Authors:** Zouaoui Setifi, Fatima Setifi, Necmi Dege, Mohammed Hadi Al-Douh, Christopher Glidewell

**Affiliations:** a Département de Technologie, Faculté de Technologie, Université 20 Août 1955-Skikda, BP 26, Route d’El-Hadaiek, Skikda 21000, Algeria; b Laboratoire de Chimie, Ingénierie Moléculaire et Nanostructures (LCIMN), Université Ferhat Abbas Sétif 1, Sétif 19000, Algeria; c Ondokuz Mayıs University, Arts and Sciences Faculty, Department of Physics, 55139 Atakum-Samsun, Turkey; d Chemistry Department, Faculty of Science, Hadhramout University, Mukalla, Hadhramout, Yemen; e School of Chemistry, University of St Andrews, St Andrews, Fife KY16 9ST, UK; University Koblenz-Landau, Germany

**Keywords:** synthesis, crystal structure, mol­ecular structure, disorder

## Abstract

In the title compound, [Fe{(C_5_H_4_N)_3_N}_2_][C{C(CN)_2_}_3_], both ions lie across centres of inversion, with the anion being statistically disordered over two sets of atomic sites having equal occupancy. The cation and anion have approximate  and 32 symmetry, respectively, and the Fe—N bond lengths indicate low-spin Fe^II^. A combination of two-centre C—H⋯N and three-centre C—H⋯(N)_2_ hydrogen bonds link the ions into complex sheets. Several low-occupancy water mol­ecules are present, whose H atoms could not be located: accordingly, the reflection data were subjected to the SQUEEZE procedure [Spek (2015 ▸). *Acta Cryst.* C**71**, 9–18].

## Abstract 2

Both ions in the title compound lie across centres of inversion, with the anion being statistically disordered.

## Graphical abstract

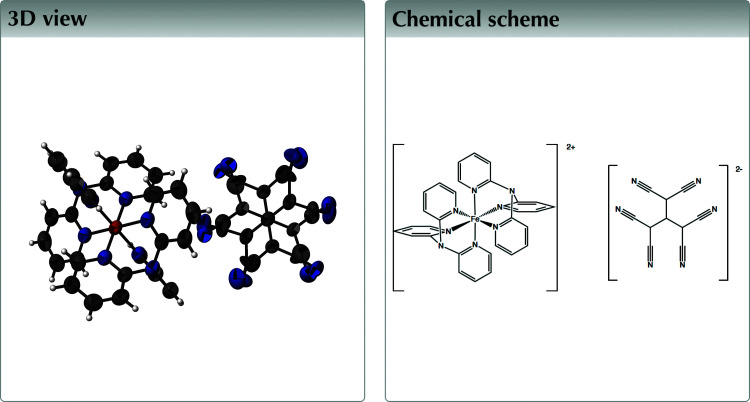

## Structure description

As a consequence of their ability to link metal ions in a variety of different ways, polynitrile anions, either functioning alone or in combination with neutral co-ligands, provide opportunities for the generation of mol­ecular architectures with varying dimensions and topologies (Miyazaki *et al.*, 2003 ▸; Benmansour *et al.*, 2007 ▸, 2008 ▸, 2012 ▸; Atmani *et al.*, 2008 ▸; Yuste *et al.*, 2009 ▸). The presence of other potential donor groups such as those derived from –OH, –SH or –NH_2_, together with their rigidity and electronic delocalization, mean that polynitrile anions can also lead to new bis­table materials (Benmansour *et al.*, 2010 ▸; Setifi *et al.*, 2009 ▸, 2014 ▸; Pittala *et al.*, 2017 ▸). As a part of our continuing study of the structural and magnetic properties of iron(II) complexes containing both polynitrile and polypyridyl units (Setifi *et al.*, 2013 ▸, 2017 ▸, 2018*a* ▸,*b* ▸), we report here the mol­ecular and supra­molecular structure of a new compound based on tri(2-pyrid­yl)amine (tpa) as ligand and the tris­(di­cyano­methyl­ene)methane­diide dianion (tcpd^2−^) as the counter-ion.

**Figure 1:**
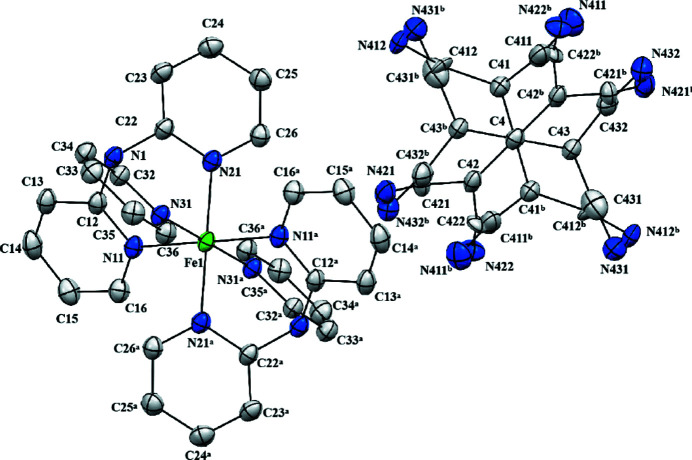
The structure of the two ionic components, showing the atom-labelling scheme. Displacement ellipsoids are drawn at the 30% probability level. The anion is disordered across a centre of inversion and the atoms marked ‘a′ or ‘b′ are at the symmetry positions (1 − *x*, 1 − *y*, 1 − *z*) and (1 − *x*, 2 − *y*, −*z*), respectively.

The structure consists of a [Fe((C_5_H_4_N)_3_N)_2_]^2+^ cation containing six-coordinate Fe in an octa­hedral coordination environment and a [C(C(CN)_2_)_3_]^2−^ anion (Fig. 1 ▸). In addition, there are also partial-occupancy water mol­ecules present, but these could not be structurally characterized in a satisfactory manner. The cation lies across a centre of inversion (½, ½, ½) with the unique ligand coordinated in a tripodal fashion, such that the point symmetry of the cation approximates very closely to *S*_6_ ( 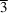 ). The Fe—N distances lie in the range 1.981 (3)–1.997 (3) Å. This is typical for six-coordinate low-spin Fe^II^ complexes, whereas Fe—N distances in analogous high-spin Fe^II^ complexes are typically observed at around 2.15 Å (Orpen *et al.*, 1989 ▸). The trigonal anion is disordered across another centre of inversion (½, 1, 0). The geometry at the central atom C4 is exactly planar, but the three independent C(CN)_2_ groups are twisted out of this plane, making dihedral angles with it of 26.2 (9), 27.7 (13) and 29.3 (9)°, so that the point symmetry of the anion approximates very closely to *D*_3_ (32). The anion is chiral, but the inversion symmetry confirms that equal numbers of the two enanti­omeric conformations are present.

**Figure 2:**
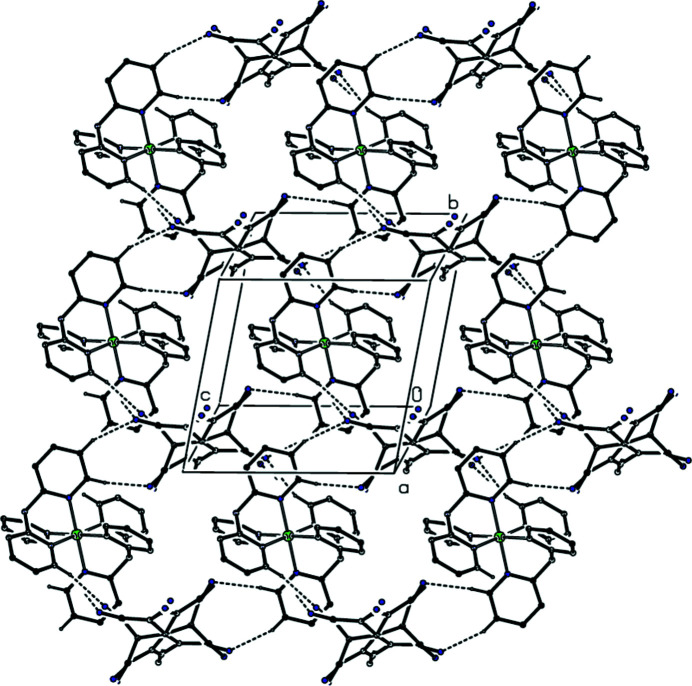
Part of the crystal structure showing the formation of a hydrogen-bonded sheet lying parallel to (100). Hydrogen bonds are drawn as dashed lines and, for the sake of clarity, H atoms not involved in the motif shown have been omitted. Each anion site is occupied by one of the two possible orientations of the anion, distributed at random.

Within the selected asymmetric unit, the cation is linked to both orientations of the disordered anion by one two-centre C—H⋯N hydrogen bond and one three-centre C—H⋯(N)_2_ hydrogen bond (Table 1 ▸), forming an ion pair. An additional further three-centre system links these ion pairs into complex sheets lying parallel to (100) (Fig. 2 ▸): within this sheet, each anion site is occupied by one of the two possible orientations of the anion, and these orientations are distributed at random throughout the structure such that equal numbers of the two exist in the crystal as a whole.

## Synthesis and crystallization

The title compound was synthesized solvothermally under autogenous pressure using a mixture of iron(II) sulfate hepta­hydrate (28 mg, 0.1 mmol), tri(2-pyrid­yl)amine (31 mg, 0.1 mmol) and dipotassium tris­(dicycano­methyl­ene)methane­diide (28 mg, 0.1 mmol) in water-ethanol (3:1 *v*/*v*, 20 ml). The mixture was sealed in a Teflon-lined autoclave and held at 423 K for 3 d, and then cooled to ambient temperature at a rate of 10 K per hour (yield 45%). Red needles of the title complex suitable for single-crystal X-ray diffraction were selected directly from the synthesized product.

## Refinement

Crystal data, data collection and structure refinement details are summarized in Table 2 ▸. Because of the extensive overlapping of the atomic sites in the disordered anion, it was found necessary to restrain the bonded C—C and C—N distances in the anion to values of 1.42 (2) and 1.16 (2) Å, respectively, while the 1,3 non-bonded C⋯N distances were restrained to 2.58 (4) Å. Conventional refinement then indicated the presence of several low-occupancy water mol­ecules, whose H atoms could not be located: accordingly, the reflection data were subjected to the SQUEEZE procedure (Spek, 2015 ▸), which indicated a void volume of 149 Å^3^ centred at the origin, and a total of 11 electrons per unit cell in addition to those of the ionic components.

## Supplementary Material

Crystal structure: contains datablock(s) global, I. DOI: 10.1107/S241431462001278X/im4010sup1.cif

Structure factors: contains datablock(s) I. DOI: 10.1107/S241431462001278X/im4010Isup2.hkl

CCDC reference: 2032866

Additional supporting information:  crystallographic information; 3D view; checkCIF report

## Figures and Tables

**Table 1:** Hydrogen-bond geometry (Å, °)

*D*—H⋯*A*	*D*—H	H⋯*A*	*D*⋯*A*	*D*—H⋯*A*
C25—H25⋯N412	0.93	2.52	3.404 (16)	159
C26—H26⋯N421	0.93	2.31	3.23 (3)	170
C26—H26⋯N432^i^	0.93	2.54	3.47 (3)	173
C36—H36⋯N412^ii^	0.93	2.48	3.352 (17)	157
C36—H36⋯N431^iii^	0.93	2.50	3.39 (2)	160

Symmetry codes: (i) 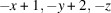 ; (ii) 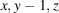 ; (iii) 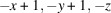 .

**Table 2:** Experimental details

Crystal data	
Chemical formula	[Fe(C_15_H_12_N_4_)_2_](C_10_N_6_)
*M*_r_	756.58
Crystal system, space group	Triclinic, *P* 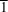
Temperature (K)	296
*a*, *b*, *c* (Å)	9.8291 (8), 10.0499 (8), 11.0308 (9)
α, β, γ (°)	98.825 (7), 90.900 (7), 117.747 (6)
*V* (Å^3^)	948.18 (14)
*Z*	1
Radiation type	Mo *K*α
μ (mm^−1^)	0.45
Crystal size (mm)	0.39 × 0.12 × 0.11

Data collection	
Diffractometer	Stoe IPDS 2
Absorption correction	Integration (*X-RED32*; Stoe & Cie, 2002 ▸)
*T*_min_, *T*_max_	0.899, 0.952
No. of measured, independent and observed [*I* > 2σ(*I*)] reflections	9558, 3955, 2609
*R*_int_	0.103
(sin θ/λ)_max_ (Å^−1^)	0.629

Refinement	
*R*[*F* ^2^ > 2σ(*F* ^2^)], *wR*(*F* ^2^), *S*	0.067, 0.164, 0.96
No. of reflections	3955
No. of parameters	319
No. of restraints	21
H-atom treatment	H-atom parameters constrained
Δρ_max_, Δρ_min_ (e Å^−3^)	1.01, −0.25

Computer programs: *X-AREA* and *X-RED32* (Stoe & Cie, 2002 ▸), *SHELXS86* (Sheldrick, 2015 ▸), *SHELXL2014* (Sheldrick, 2015 ▸), *Mercury* (Macrae *et al.*, 2020 ▸) and *PLATON* (Spek, 2020 ▸).

## full crystallographic data

### Crystal data

**Table:**

[Fe(C_15_H_12_N_4_)_2_](C_10_N_6_)	*Z* = 1
*M**_r_* = 756.58	*F*(000) = 388
Triclinic, *P*1	*D*_x_ = 1.325 Mg m^−^^3^
*a* = 9.8291 (8) Å	Mo *K*α radiation, λ = 0.71073 Å
*b* = 10.0499 (8) Å	Cell parameters from 4139 reflections
*c* = 11.0308 (9) Å	θ = 1.9–27.1°
α = 98.825 (7)°	µ = 0.45 mm^−^^1^
β = 90.900 (7)°	*T* = 296 K
γ = 117.747 (6)°	Needle, red
*V* = 948.18 (14) Å^3^	0.39 × 0.12 × 0.11 mm

### Data collection

**Table:**

STOE IPDS 2 diffractometer	3955 independent reflections
Radiation source: sealed X-ray tube, 12 x 0.4 mm long-fine focus	2609 reflections with *I* > 2σ(*I*)
Graphite monochromator	*R*_int_ = 0.103
rotation method scans	θ_max_ = 26.6°, θ_min_ = 1.9°
Absorption correction: integration (X-RED32; Stoe & Cie, 2002)	*h* = −12→12
*T*_min_ = 0.899, *T*_max_ = 0.952	*k* = −12→12
9558 measured reflections	*l* = −13→13

### Refinement

**Table:**

Refinement on *F*^2^	Primary atom site location: difference Fourier map
Least-squares matrix: full	Hydrogen site location: inferred from neighbouring sites
*R*[*F*^2^ > 2σ(*F*^2^)] = 0.067	H-atom parameters constrained
*wR*(*F*^2^) = 0.164	*w* = 1/[σ^2^(*F*_o_^2^) + (0.0793*P*)^2^] where *P* = (*F*_o_^2^ + 2*F*_c_^2^)/3
*S* = 0.96	(Δ/σ)_max_ < 0.001
3955 reflections	Δρ_max_ = 1.01 e Å^−^^3^
319 parameters	Δρ_min_ = −0.25 e Å^−^^3^
21 restraints	

### Special details

**Table:**

Geometry. All esds (except the esd in the dihedral angle between two l.s. planes) are estimated using the full covariance matrix. The cell esds are taken into account individually in the estimation of esds in distances, angles and torsion angles; correlations between esds in cell parameters are only used when they are defined by crystal symmetry. An approximate (isotropic) treatment of cell esds is used for estimating esds involving l.s. planes.

### Fractional atomic coordinates and isotropic or equivalent isotropic displacement parameters (Å^2^)

**Table:**

	*x*	*y*	*z*	*U*_iso_*/*U*_eq_	Occ. (<1)
Fe1	0.5000	0.5000	0.5000	0.0477 (2)	
N1	0.6353 (4)	0.6637 (3)	0.7523 (3)	0.0559 (7)	
N11	0.3931 (4)	0.4694 (4)	0.6537 (3)	0.0543 (7)	
C12	0.4770 (5)	0.5581 (4)	0.7590 (3)	0.0547 (9)	
C13	0.4143 (6)	0.5505 (5)	0.8704 (4)	0.0666 (11)	
H13	0.4751	0.6135	0.9422	0.080*	
C14	0.2641 (6)	0.4510 (6)	0.8747 (4)	0.0801 (13)	
H14	0.2197	0.4468	0.9487	0.096*	
C15	0.1770 (5)	0.3547 (6)	0.7664 (4)	0.0752 (12)	
H15	0.0743	0.2826	0.7671	0.090*	
C16	0.2460 (5)	0.3686 (5)	0.6582 (4)	0.0648 (10)	
H16	0.1878	0.3053	0.5855	0.078*	
N21	0.5931 (3)	0.7238 (3)	0.5604 (3)	0.0541 (7)	
C22	0.6498 (4)	0.7761 (4)	0.6797 (3)	0.0542 (8)	
C23	0.7155 (5)	0.9278 (4)	0.7338 (4)	0.0641 (10)	
H23	0.7532	0.9593	0.8167	0.077*	
C24	0.7241 (5)	1.0313 (5)	0.6631 (4)	0.0716 (11)	
H24	0.7671	1.1347	0.6972	0.086*	
C25	0.6677 (5)	0.9800 (5)	0.5398 (4)	0.0686 (11)	
H25	0.6731	1.0489	0.4902	0.082*	
C26	0.6037 (5)	0.8267 (5)	0.4910 (4)	0.0608 (9)	
H26	0.5669	0.7934	0.4079	0.073*	
N31	0.6765 (3)	0.5035 (3)	0.5924 (3)	0.0513 (7)	
C32	0.7226 (4)	0.5871 (4)	0.7074 (3)	0.0512 (8)	
C33	0.8442 (5)	0.6016 (5)	0.7805 (4)	0.0635 (10)	
H33	0.8723	0.6618	0.8588	0.076*	
C34	0.9249 (5)	0.5253 (5)	0.7360 (4)	0.0685 (11)	
H34	1.0080	0.5323	0.7832	0.082*	
C35	0.8768 (5)	0.4382 (5)	0.6184 (4)	0.0644 (10)	
H35	0.9279	0.3850	0.5858	0.077*	
C36	0.7564 (4)	0.4297 (4)	0.5503 (4)	0.0578 (9)	
H36	0.7274	0.3708	0.4715	0.069*	
C4	0.5000	1.0000	0.0000	0.0528 (12)	
C41	0.6447 (8)	1.1099 (8)	0.0634 (6)	0.0546 (17)	0.5
C42	0.4036 (9)	0.8718 (8)	0.0533 (6)	0.0577 (19)	0.5
C43	0.4494 (9)	1.0165 (9)	−0.1150 (6)	0.0536 (17)	0.5
C411	0.768 (3)	1.194 (3)	−0.001 (3)	0.081 (9)	0.5
N411	0.867 (2)	1.287 (2)	−0.042 (2)	0.093 (6)	0.5
C412	0.673 (2)	1.129 (2)	0.1959 (11)	0.053 (4)	0.5
N412	0.704 (3)	1.168 (2)	0.3007 (12)	0.078 (4)	0.5
C421	0.462 (4)	0.816 (3)	0.142 (3)	0.057 (5)	0.5
N421	0.508 (3)	0.755 (2)	0.198 (3)	0.088 (7)	0.5
C422	0.2396 (13)	0.790 (3)	0.024 (2)	0.054 (5)	0.5
N422	0.1088 (13)	0.7435 (18)	0.0259 (19)	0.073 (3)	0.5
C431	0.353 (3)	0.888 (3)	−0.204 (2)	0.100 (10)	0.5
N431	0.267 (4)	0.791 (3)	−0.2778 (18)	0.095 (6)	0.5
C432	0.499 (4)	1.164 (3)	−0.145 (3)	0.074 (10)	0.5
N432	0.545 (3)	1.275 (3)	−0.184 (3)	0.086 (6)	0.5

### Atomic displacement parameters (Å^2^)

**Table:**

	*U*^11^	*U*^22^	*U*^33^	*U*^12^	*U*^13^	*U*^23^
Fe1	0.0555 (5)	0.0561 (5)	0.0382 (4)	0.0336 (4)	0.0043 (3)	0.0028 (3)
N1	0.070 (2)	0.0617 (18)	0.0440 (16)	0.0404 (17)	0.0011 (14)	0.0008 (14)
N11	0.0626 (19)	0.0666 (19)	0.0452 (16)	0.0405 (17)	0.0106 (14)	0.0082 (14)
C12	0.071 (2)	0.062 (2)	0.0450 (19)	0.043 (2)	0.0107 (16)	0.0086 (17)
C13	0.089 (3)	0.081 (3)	0.048 (2)	0.054 (3)	0.016 (2)	0.0118 (19)
C14	0.103 (4)	0.100 (3)	0.066 (3)	0.066 (3)	0.038 (3)	0.033 (3)
C15	0.072 (3)	0.091 (3)	0.079 (3)	0.047 (3)	0.027 (2)	0.030 (3)
C16	0.065 (3)	0.077 (3)	0.060 (2)	0.039 (2)	0.0115 (19)	0.014 (2)
N21	0.0614 (19)	0.0607 (18)	0.0473 (16)	0.0364 (16)	0.0050 (13)	0.0050 (14)
C22	0.061 (2)	0.061 (2)	0.0462 (19)	0.0361 (18)	0.0027 (16)	0.0019 (17)
C23	0.073 (3)	0.061 (2)	0.059 (2)	0.037 (2)	−0.0034 (19)	−0.0055 (19)
C24	0.079 (3)	0.061 (2)	0.075 (3)	0.038 (2)	0.005 (2)	−0.001 (2)
C25	0.080 (3)	0.064 (2)	0.070 (3)	0.040 (2)	0.009 (2)	0.016 (2)
C26	0.066 (2)	0.067 (2)	0.057 (2)	0.037 (2)	0.0058 (18)	0.0144 (19)
N31	0.0567 (17)	0.0574 (17)	0.0448 (15)	0.0334 (15)	0.0038 (12)	0.0022 (13)
C32	0.056 (2)	0.057 (2)	0.0467 (19)	0.0323 (17)	0.0010 (15)	0.0046 (16)
C33	0.070 (3)	0.069 (2)	0.053 (2)	0.036 (2)	−0.0039 (18)	0.0051 (19)
C34	0.060 (2)	0.078 (3)	0.072 (3)	0.037 (2)	−0.0026 (19)	0.015 (2)
C35	0.063 (2)	0.076 (3)	0.069 (3)	0.045 (2)	0.0118 (19)	0.013 (2)
C36	0.065 (2)	0.065 (2)	0.053 (2)	0.040 (2)	0.0092 (17)	0.0065 (18)
C4	0.062 (3)	0.060 (3)	0.043 (3)	0.036 (3)	0.006 (2)	0.001 (2)
C41	0.056 (4)	0.060 (4)	0.047 (4)	0.028 (4)	0.003 (4)	0.005 (4)
C42	0.080 (6)	0.064 (5)	0.038 (4)	0.043 (4)	0.002 (3)	0.001 (3)
C43	0.059 (4)	0.060 (5)	0.042 (4)	0.030 (4)	−0.001 (3)	0.004 (4)
C411	0.119 (19)	0.065 (13)	0.058 (10)	0.046 (11)	−0.005 (8)	−0.001 (8)
N411	0.124 (15)	0.088 (9)	0.099 (9)	0.073 (10)	0.023 (8)	0.029 (6)
C412	0.054 (7)	0.057 (7)	0.033 (6)	0.023 (5)	−0.006 (5)	−0.022 (5)
N412	0.094 (12)	0.084 (11)	0.034 (5)	0.026 (8)	−0.007 (7)	0.000 (6)
C421	0.071 (13)	0.069 (11)	0.050 (8)	0.045 (9)	0.013 (7)	0.022 (7)
N421	0.15 (2)	0.071 (8)	0.059 (7)	0.065 (12)	−0.008 (11)	0.010 (8)
C422	0.044 (7)	0.042 (6)	0.064 (10)	0.007 (6)	0.025 (7)	0.020 (6)
N422	0.052 (6)	0.066 (8)	0.092 (8)	0.022 (6)	0.018 (5)	0.013 (6)
C431	0.078 (13)	0.117 (18)	0.12 (2)	0.052 (12)	0.016 (11)	0.050 (15)
N431	0.099 (11)	0.085 (12)	0.072 (12)	0.022 (9)	0.006 (9)	0.010 (8)
C432	0.078 (16)	0.085 (12)	0.053 (9)	0.033 (9)	0.019 (9)	0.009 (9)
N432	0.097 (12)	0.094 (12)	0.061 (10)	0.038 (9)	0.007 (8)	0.019 (9)

### Geometric parameters (Å, º)

**Table:**

Fe1—N31^i^	1.981 (3)	N31—C32	1.349 (4)
Fe1—N31	1.981 (3)	N31—C36	1.353 (4)
Fe1—N21^i^	1.986 (3)	C32—C33	1.368 (5)
Fe1—N21	1.986 (3)	C33—C34	1.386 (6)
Fe1—N11^i^	1.997 (3)	C33—H33	0.9300
Fe1—N11	1.997 (3)	C34—C35	1.386 (6)
N1—C12	1.430 (5)	C34—H34	0.9300
N1—C22	1.438 (5)	C35—C36	1.353 (5)
N1—C32	1.444 (4)	C35—H35	0.9300
N11—C16	1.331 (5)	C36—H36	0.9300
N11—C12	1.339 (5)	C4—C41^ii^	1.414 (7)
C12—C13	1.380 (5)	C4—C41	1.414 (7)
C13—C14	1.350 (7)	C4—C43	1.416 (7)
C13—H13	0.9300	C4—C43^ii^	1.416 (7)
C14—C15	1.392 (7)	C4—C42	1.420 (7)
C14—H14	0.9300	C4—C42^ii^	1.420 (7)
C15—C16	1.376 (6)	C41—C411	1.395 (15)
C15—H15	0.9300	C41—C412	1.450 (14)
C16—H16	0.9300	C42—C422	1.431 (14)
N21—C26	1.346 (5)	C42—C421	1.435 (16)
N21—C22	1.346 (4)	C43—C431	1.402 (16)
C22—C23	1.374 (5)	C43—C432	1.426 (16)
C23—C24	1.367 (6)	C411—N411	1.147 (17)
C23—H23	0.9300	C412—N412	1.150 (15)
C24—C25	1.385 (6)	C421—N421	1.149 (16)
C24—H24	0.9300	C422—N422	1.147 (14)
C25—C26	1.375 (6)	C431—N431	1.138 (17)
C25—H25	0.9300	C432—N432	1.147 (17)
C26—H26	0.9300		
			
N31^i^—Fe1—N31	180.00 (17)	C431^ii^—C41—C422^ii^	135.0 (14)
N31^i^—Fe1—N21^i^	88.18 (12)	C4—C42—C43^ii^	59.7 (4)
N31—Fe1—N21^i^	91.82 (11)	C4—C42—C422	122.3 (11)
N31^i^—Fe1—N21	91.82 (12)	C43^ii^—C42—C422	159.3 (13)
N31—Fe1—N21	88.18 (12)	C4—C42—C41^ii^	59.5 (4)
N21^i^—Fe1—N21	180.00 (10)	C43^ii^—C42—C41^ii^	119.1 (7)
N31^i^—Fe1—N11^i^	87.54 (12)	C422—C42—C41^ii^	67.0 (10)
N31—Fe1—N11^i^	92.46 (12)	C4—C42—C421	122.9 (14)
N21^i^—Fe1—N11^i^	87.97 (12)	C43^ii^—C42—C421	66.9 (12)
N21—Fe1—N11^i^	92.03 (12)	C422—C42—C421	114.8 (17)
N31^i^—Fe1—N11	92.46 (12)	C41^ii^—C42—C421	159.9 (16)
N31—Fe1—N11	87.55 (12)	C4—C42—C411^ii^	110.2 (10)
N21^i^—Fe1—N11	92.03 (12)	C43^ii^—C42—C411^ii^	157.1 (15)
N21—Fe1—N11	87.97 (12)	C41^ii^—C42—C411^ii^	55.5 (9)
N11^i^—Fe1—N11	180.00 (18)	C421—C42—C411^ii^	126.9 (16)
C12—N1—C22	111.5 (3)	C4—C42—C432^ii^	111.2 (11)
C12—N1—C32	112.0 (3)	C43^ii^—C42—C432^ii^	56.5 (9)
C22—N1—C32	111.1 (3)	C422—C42—C432^ii^	126.6 (15)
C16—N11—C12	118.5 (3)	C41^ii^—C42—C432^ii^	155.9 (16)
C16—N11—Fe1	124.8 (3)	C411^ii^—C42—C432^ii^	138.6 (14)
C12—N11—Fe1	116.8 (3)	C41^ii^—C43—C431	67.3 (15)
N11—C12—C13	121.9 (4)	C41^ii^—C43—C4	60.5 (4)
N11—C12—N1	117.5 (3)	C431—C43—C4	121.2 (14)
C13—C12—N1	120.6 (4)	C41^ii^—C43—C432	155.1 (18)
C14—C13—C12	119.7 (4)	C431—C43—C432	118 (2)
C14—C13—H13	120.2	C4—C43—C432	120.7 (15)
C12—C13—H13	120.2	C41^ii^—C43—C42^ii^	120.4 (6)
C13—C14—C15	118.9 (4)	C431—C43—C42^ii^	153.4 (14)
C13—C14—H14	120.5	C4—C43—C42^ii^	59.9 (4)
C15—C14—H14	120.5	C432—C43—C42^ii^	66.9 (13)
C16—C15—C14	118.6 (4)	C41^ii^—C43—C412^ii^	59.6 (8)
C16—C15—H15	120.7	C4—C43—C412^ii^	116.2 (9)
C14—C15—H15	120.7	C432—C43—C412^ii^	122.8 (16)
N11—C16—C15	122.4 (4)	C42^ii^—C43—C412^ii^	159.8 (10)
N11—C16—H16	118.8	C41^ii^—C43—C421^ii^	162.5 (15)
C15—C16—H16	118.8	C431—C43—C421^ii^	124.0 (17)
C26—N21—C22	117.7 (3)	C4—C43—C421^ii^	113.7 (11)
C26—N21—Fe1	124.9 (3)	C42^ii^—C43—C421^ii^	56.8 (10)
C22—N21—Fe1	117.4 (2)	C412^ii^—C43—C421^ii^	130.0 (13)
N21—C22—C23	123.4 (3)	C422^ii^—C411—N422^ii^	83 (4)
N21—C22—N1	116.6 (3)	C422^ii^—C411—N411	59 (4)
C23—C22—N1	119.9 (3)	C422^ii^—C411—C41	117 (6)
C24—C23—C22	118.5 (4)	N422^ii^—C411—C41	159 (3)
C24—C23—H23	120.7	N411—C411—C41	167 (4)
C22—C23—H23	120.7	C422^ii^—C411—C42^ii^	62 (5)
C23—C24—C25	118.9 (4)	N422^ii^—C411—C42^ii^	142 (3)
C23—C24—H24	120.5	N411—C411—C42^ii^	121 (3)
C25—C24—H24	120.5	C41—C411—C42^ii^	57.7 (9)
C26—C25—C24	119.8 (4)	N422^ii^—N411—C422^ii^	91 (3)
C26—C25—H25	120.1	N422^ii^—N411—C411	76 (3)
C24—C25—H25	120.1	C431^ii^—C412—N431^ii^	101 (6)
N21—C26—C25	121.6 (4)	C431^ii^—C412—N412	79 (7)
N21—C26—H26	119.2	C431^ii^—C412—C41	108 (7)
C25—C26—H26	119.2	N431^ii^—C412—C41	145 (2)
C32—N31—C36	116.5 (3)	N412—C412—C41	169 (2)
C32—N31—Fe1	117.7 (2)	C431^ii^—C412—C43^ii^	59 (5)
C36—N31—Fe1	125.8 (2)	N431^ii^—C412—C43^ii^	158 (2)
N31—C32—C33	123.8 (3)	N412—C412—C43^ii^	134 (2)
N31—C32—N1	116.2 (3)	C41—C412—C43^ii^	55.9 (5)
C33—C32—N1	120.0 (3)	N431^ii^—N412—C431^ii^	79 (4)
C32—C33—C34	119.0 (4)	N431^ii^—N412—C412	67 (3)
C32—C33—H33	120.5	C432^ii^—C421—N432^ii^	96 (5)
C34—C33—H33	120.5	C432^ii^—C421—N421	73 (5)
C35—C34—C33	117.3 (4)	C432^ii^—C421—C42	108 (6)
C35—C34—H34	121.3	N432^ii^—C421—C42	151 (3)
C33—C34—H34	121.3	N421—C421—C42	169 (4)
C36—C35—C34	120.8 (3)	C432^ii^—C421—C43^ii^	58 (5)
C36—C35—H35	119.6	N432^ii^—C421—C43^ii^	152 (3)
C34—C35—H35	119.6	N421—C421—C43^ii^	128 (3)
C35—C36—N31	122.6 (4)	C42—C421—C43^ii^	56.3 (7)
C35—C36—H36	118.7	N432^ii^—N421—C432^ii^	84 (4)
N31—C36—H36	118.7	N432^ii^—N421—C421	67 (4)
C41^ii^—C4—C41	180.0 (5)	C411^ii^—C422—N411^ii^	104 (6)
C41^ii^—C4—C43	58.9 (5)	C411^ii^—C422—N422	80 (5)
C41—C4—C43	121.1 (5)	C411^ii^—C422—C42	106 (5)
C41^ii^—C4—C43^ii^	121.1 (5)	N411^ii^—C422—C42	150 (3)
C41—C4—C43^ii^	58.9 (5)	N422—C422—C42	164 (3)
C43—C4—C43^ii^	180.0 (7)	C411^ii^—C422—C41^ii^	52 (4)
C41^ii^—C4—C42	60.7 (4)	N411^ii^—C422—C41^ii^	153 (3)
C41—C4—C42	119.3 (4)	N422—C422—C41^ii^	131 (2)
C43—C4—C42	119.6 (4)	C42—C422—C41^ii^	56.5 (5)
C43^ii^—C4—C42	60.4 (4)	N411^ii^—N422—C411^ii^	78 (3)
C41^ii^—C4—C42^ii^	119.3 (4)	N411^ii^—N422—C422	62 (3)
C41—C4—C42^ii^	60.7 (4)	C412^ii^—C431—N412^ii^	88 (6)
C43—C4—C42^ii^	60.4 (4)	C412^ii^—C431—N431	67 (5)
C43^ii^—C4—C42^ii^	119.6 (4)	C412^ii^—C431—C43	112 (6)
C42—C4—C42^ii^	180.0	N412^ii^—C431—C43	152 (3)
C43^ii^—C41—C411	154.6 (16)	N431—C431—C43	174 (4)
C43^ii^—C41—C4	60.6 (4)	C412^ii^—C431—C41^ii^	63 (7)
C411—C41—C4	120.8 (14)	N412^ii^—C431—C41^ii^	150 (3)
C43^ii^—C41—C42^ii^	120.5 (7)	N431—C431—C41^ii^	126 (3)
C411—C41—C42^ii^	66.8 (14)	C43—C431—C41^ii^	56.1 (9)
C4—C41—C42^ii^	59.9 (4)	N412^ii^—N431—C412^ii^	87 (4)
C43^ii^—C41—C412	64.6 (9)	N412^ii^—N431—C431	76 (4)
C411—C41—C412	118.0 (16)	C421^ii^—C432—N421^ii^	90 (5)
C4—C41—C412	120.9 (10)	C421^ii^—C432—N432	67 (5)
C42^ii^—C41—C412	159.6 (10)	C421^ii^—C432—C43	111 (6)
C43^ii^—C41—C431^ii^	56.7 (10)	N421^ii^—C432—C43	154 (3)
C411—C41—C431^ii^	127.1 (17)	N432—C432—C43	170 (4)
C4—C41—C431^ii^	111.9 (11)	C421^ii^—C432—C42^ii^	60 (6)
C42^ii^—C41—C431^ii^	156.5 (12)	N421^ii^—C432—C42^ii^	149 (3)
C43^ii^—C41—C422^ii^	160.3 (12)	N432—C432—C42^ii^	125 (3)
C4—C41—C422^ii^	112.9 (9)	C43—C432—C42^ii^	56.6 (8)
C42^ii^—C41—C422^ii^	56.5 (8)	N421^ii^—N432—C421^ii^	88 (4)
C412—C41—C422^ii^	126.1 (13)	N421^ii^—N432—C432	72 (4)
			
C16—N11—C12—C13	−1.7 (5)	C412—C41—C411—C42^ii^	−158.0 (11)
Fe1—N11—C12—C13	178.1 (3)	C431^ii^—C41—C411—C42^ii^	−158.0 (16)
C16—N11—C12—N1	178.9 (3)	C422^ii^—C41—C411—C42^ii^	−19 (12)
Fe1—N11—C12—N1	−1.3 (4)	C422^ii^—C411—N411—N422^ii^	−154 (12)
C22—N1—C12—N11	63.6 (4)	C41—C411—N411—N422^ii^	128 (13)
C32—N1—C12—N11	−61.7 (4)	C42^ii^—C411—N411—N422^ii^	−150 (5)
C22—N1—C12—C13	−115.8 (4)	N422^ii^—C411—N411—C422^ii^	154 (12)
C32—N1—C12—C13	118.9 (3)	C41—C411—N411—C422^ii^	−78 (17)
N11—C12—C13—C14	0.1 (6)	C42^ii^—C411—N411—C422^ii^	4 (9)
N1—C12—C13—C14	179.4 (3)	C43^ii^—C41—C412—C431^ii^	−28 (11)
C12—C13—C14—C15	2.0 (6)	C411—C41—C412—C431^ii^	−180 (11)
C13—C14—C15—C16	−2.3 (6)	C4—C41—C412—C431^ii^	−5 (11)
C12—N11—C16—C15	1.3 (5)	C42^ii^—C41—C412—C431^ii^	81 (11)
Fe1—N11—C16—C15	−178.4 (3)	C422^ii^—C41—C412—C431^ii^	171 (10)
C14—C15—C16—N11	0.7 (6)	C43^ii^—C41—C412—N431^ii^	−171 (5)
C26—N21—C22—C23	0.9 (5)	C411—C41—C412—N431^ii^	38 (5)
Fe1—N21—C22—C23	−179.2 (3)	C4—C41—C412—N431^ii^	−147 (4)
C26—N21—C22—N1	179.3 (3)	C42^ii^—C41—C412—N431^ii^	−61 (6)
Fe1—N21—C22—N1	−0.8 (4)	C431^ii^—C41—C412—N431^ii^	−142 (13)
C12—N1—C22—N21	−62.2 (4)	C422^ii^—C41—C412—N431^ii^	29 (5)
C32—N1—C22—N21	63.6 (4)	C43^ii^—C41—C412—N412	−156 (15)
C12—N1—C22—C23	116.3 (4)	C411—C41—C412—N412	52 (15)
C32—N1—C22—C23	−118.0 (4)	C4—C41—C412—N412	−133 (14)
N21—C22—C23—C24	−0.1 (6)	C42^ii^—C41—C412—N412	−47 (16)
N1—C22—C23—C24	−178.4 (4)	C431^ii^—C41—C412—N412	−128 (23)
C22—C23—C24—C25	−0.5 (6)	C422^ii^—C41—C412—N412	43 (15)
C23—C24—C25—C26	0.3 (7)	C411—C41—C412—C43^ii^	−151.7 (19)
C22—N21—C26—C25	−1.1 (5)	C4—C41—C412—C43^ii^	23.3 (9)
Fe1—N21—C26—C25	179.0 (3)	C42^ii^—C41—C412—C43^ii^	109 (3)
C24—C25—C26—N21	0.5 (6)	C431^ii^—C41—C412—C43^ii^	28 (11)
C36—N31—C32—C33	0.8 (5)	C422^ii^—C41—C412—C43^ii^	−160.6 (15)
Fe1—N31—C32—C33	−178.8 (3)	C431^ii^—C412—N412—N431^ii^	−150 (13)
C36—N31—C32—N1	−179.1 (3)	C41—C412—N412—N431^ii^	−20 (21)
Fe1—N31—C32—N1	1.2 (4)	C43^ii^—C412—N412—N431^ii^	−172 (7)
C12—N1—C32—N31	61.7 (4)	N431^ii^—C412—N412—C431^ii^	150 (13)
C22—N1—C32—N31	−63.8 (4)	C41—C412—N412—C431^ii^	130 (22)
C12—N1—C32—C33	−118.3 (4)	C43^ii^—C412—N412—C431^ii^	−22 (9)
C22—N1—C32—C33	116.2 (4)	C4—C42—C421—C432^ii^	−6 (15)
N31—C32—C33—C34	−0.9 (6)	C43^ii^—C42—C421—C432^ii^	−27 (13)
N1—C32—C33—C34	179.1 (4)	C422—C42—C421—C432^ii^	176 (13)
C32—C33—C34—C35	0.2 (6)	C41^ii^—C42—C421—C432^ii^	85 (13)
C33—C34—C35—C36	0.4 (6)	C411^ii^—C42—C421—C432^ii^	174 (13)
C34—C35—C36—N31	−0.4 (6)	C4—C42—C421—N432^ii^	−150 (7)
C32—N31—C36—C35	−0.2 (5)	C43^ii^—C42—C421—N432^ii^	−171 (9)
Fe1—N31—C36—C35	179.5 (3)	C422—C42—C421—N432^ii^	31 (9)
C43—C4—C41—C43^ii^	179.999 (2)	C41^ii^—C42—C421—N432^ii^	−59 (11)
C42—C4—C41—C43^ii^	0.5 (6)	C411^ii^—C42—C421—N432^ii^	30 (9)
C42^ii^—C4—C41—C43^ii^	−179.5 (6)	C432^ii^—C42—C421—N432^ii^	−144 (21)
C43—C4—C41—C411	−29.3 (19)	C4—C42—C421—N421	−96 (17)
C43^ii^—C4—C41—C411	150.7 (19)	C43^ii^—C42—C421—N421	−118 (18)
C42—C4—C41—C411	151.1 (18)	C432^ii^—C42—C421—N421	−91 (26)
C42^ii^—C4—C41—C411	−28.9 (18)	C4—C42—C421—C43^ii^	21.5 (16)
C43—C4—C41—C42^ii^	−0.5 (6)	C422—C42—C421—C43^ii^	−157.4 (14)
C43^ii^—C4—C41—C42^ii^	179.5 (6)	C41^ii^—C42—C421—C43^ii^	112 (4)
C42—C4—C41—C42^ii^	179.999 (2)	C411^ii^—C42—C421—C43^ii^	−158.7 (18)
C43—C4—C41—C412	155.8 (10)	C432^ii^—C42—C421—C43^ii^	27 (13)
C43^ii^—C4—C41—C412	−24.2 (10)	C432^ii^—C421—N421—N432^ii^	−166 (16)
C42—C4—C41—C412	−23.7 (11)	C42—C421—N421—N432^ii^	−72 (21)
C42^ii^—C4—C41—C412	156.3 (11)	C43^ii^—C421—N421—N432^ii^	177 (8)
C43—C4—C41—C431^ii^	154.9 (12)	N432^ii^—C421—N421—C432^ii^	166 (16)
C43^ii^—C4—C41—C431^ii^	−25.1 (12)	C43^ii^—C421—N421—C432^ii^	−17 (10)
C42—C4—C41—C431^ii^	−24.6 (13)	C4—C42—C422—C411^ii^	6 (12)
C42^ii^—C4—C41—C431^ii^	155.4 (13)	C43^ii^—C42—C422—C411^ii^	95 (11)
C43—C4—C41—C422^ii^	−20.8 (13)	C41^ii^—C42—C422—C411^ii^	−17 (11)
C43^ii^—C4—C41—C422^ii^	159.2 (13)	C421—C42—C422—C411^ii^	−175 (11)
C42—C4—C41—C422^ii^	159.7 (12)	C432^ii^—C42—C422—C411^ii^	−174 (11)
C42^ii^—C4—C41—C422^ii^	−20.3 (12)	C4—C42—C422—N411^ii^	−167 (5)
C41^ii^—C4—C42—C43^ii^	179.5 (6)	C43^ii^—C42—C422—N411^ii^	−78 (7)
C41—C4—C42—C43^ii^	−0.5 (6)	C41^ii^—C42—C422—N411^ii^	170 (6)
C43—C4—C42—C43^ii^	180.001 (2)	C421—C42—C422—N411^ii^	12 (6)
C41^ii^—C4—C42—C422	−24.7 (14)	C411^ii^—C42—C422—N411^ii^	−173 (16)
C41—C4—C42—C422	155.3 (14)	C432^ii^—C42—C422—N411^ii^	13 (7)
C43—C4—C42—C422	−24.2 (15)	C4—C42—C422—N422	−102 (9)
C43^ii^—C4—C42—C422	155.8 (15)	C43^ii^—C42—C422—N422	−14 (12)
C41—C4—C42—C41^ii^	179.999 (2)	C41^ii^—C42—C422—N422	−125 (9)
C43—C4—C42—C41^ii^	0.5 (6)	C421—C42—C422—N422	77 (9)
C43^ii^—C4—C42—C41^ii^	−179.5 (6)	C411^ii^—C42—C422—N422	−109 (17)
C41^ii^—C4—C42—C421	156.5 (19)	C432^ii^—C42—C422—N422	78 (9)
C41—C4—C42—C421	−23.5 (19)	C4—C42—C422—C41^ii^	23.0 (13)
C43—C4—C42—C421	157.0 (18)	C43^ii^—C42—C422—C41^ii^	112 (3)
C43^ii^—C4—C42—C421	−23.0 (18)	C421—C42—C422—C41^ii^	−158.1 (17)
C41^ii^—C4—C42—C411^ii^	−23.3 (14)	C411^ii^—C42—C422—C41^ii^	17 (11)
C41—C4—C42—C411^ii^	156.7 (14)	C432^ii^—C42—C422—C41^ii^	−157 (2)
C43—C4—C42—C411^ii^	−22.8 (15)	C411^ii^—C422—N422—N411^ii^	155 (12)
C43^ii^—C4—C42—C411^ii^	157.2 (15)	C42—C422—N422—N411^ii^	−93 (10)
C41^ii^—C4—C42—C432^ii^	155.3 (17)	C41^ii^—C422—N422—N411^ii^	152 (5)
C41—C4—C42—C432^ii^	−24.7 (17)	N411^ii^—C422—N422—C411^ii^	−155 (12)
C43—C4—C42—C432^ii^	155.8 (16)	C42—C422—N422—C411^ii^	112 (16)
C43^ii^—C4—C42—C432^ii^	−24.2 (16)	C41^ii^—C422—N422—C411^ii^	−3 (8)
C41—C4—C43—C41^ii^	180.001 (3)	C41^ii^—C43—C431—C412^ii^	30 (12)
C42—C4—C43—C41^ii^	−0.5 (6)	C4—C43—C431—C412^ii^	58 (13)
C42^ii^—C4—C43—C41^ii^	179.5 (6)	C432—C43—C431—C412^ii^	−123 (12)
C41^ii^—C4—C43—C431	−30.5 (16)	C42^ii^—C43—C431—C412^ii^	142 (11)
C41—C4—C43—C431	149.5 (16)	C421^ii^—C43—C431—C412^ii^	−135 (12)
C42—C4—C43—C431	−30.9 (16)	C41^ii^—C43—C431—N412^ii^	166 (7)
C42^ii^—C4—C43—C431	149.1 (16)	C4—C43—C431—N412^ii^	−166 (6)
C41^ii^—C4—C43—C432	151 (2)	C432—C43—C431—N412^ii^	13 (7)
C41—C4—C43—C432	−29 (2)	C42^ii^—C43—C431—N412^ii^	−81 (7)
C42—C4—C43—C432	150.6 (19)	C412^ii^—C43—C431—N412^ii^	136 (16)
C42^ii^—C4—C43—C432	−29.4 (19)	C421^ii^—C43—C431—N412^ii^	1 (7)
C41^ii^—C4—C43—C42^ii^	−179.5 (6)	C4—C43—C431—C41^ii^	28.6 (13)
C41—C4—C43—C42^ii^	0.5 (6)	C432—C43—C431—C41^ii^	−153 (2)
C42—C4—C43—C42^ii^	180.001 (3)	C42^ii^—C43—C431—C41^ii^	113 (3)
C41^ii^—C4—C43—C412^ii^	−22.0 (9)	C412^ii^—C43—C431—C41^ii^	−30 (12)
C41—C4—C43—C412^ii^	158.0 (9)	C421^ii^—C43—C431—C41^ii^	−164.6 (18)
C42—C4—C43—C412^ii^	−22.5 (11)	C412^ii^—C431—N431—N412^ii^	149 (13)
C42^ii^—C4—C43—C412^ii^	157.5 (11)	C41^ii^—C431—N431—N412^ii^	172 (7)
C41^ii^—C4—C43—C421^ii^	161.5 (16)	N412^ii^—C431—N431—C412^ii^	−149 (13)
C41—C4—C43—C421^ii^	−18.5 (16)	C41^ii^—C431—N431—C412^ii^	23 (9)
C42—C4—C43—C421^ii^	161.0 (15)	C41^ii^—C43—C432—C421^ii^	141 (12)
C42^ii^—C4—C43—C421^ii^	−19.0 (15)	C431—C43—C432—C421^ii^	−123 (14)
C43^ii^—C41—C411—C422^ii^	131 (11)	C4—C43—C432—C421^ii^	55 (15)
C4—C41—C411—C422^ii^	46 (13)	C42^ii^—C43—C432—C421^ii^	28 (14)
C42^ii^—C41—C411—C422^ii^	19 (12)	C412^ii^—C43—C432—C421^ii^	−132 (14)
C412—C41—C411—C422^ii^	−139 (12)	C41^ii^—C43—C432—N421^ii^	−77 (11)
C431^ii^—C41—C411—C422^ii^	−139 (12)	C431—C43—C432—N421^ii^	19 (10)
C43^ii^—C41—C411—N422^ii^	−57 (11)	C4—C43—C432—N421^ii^	−162 (8)
C4—C41—C411—N422^ii^	−142 (9)	C42^ii^—C43—C432—N421^ii^	170 (10)
C42^ii^—C41—C411—N422^ii^	−169 (10)	C412^ii^—C43—C432—N421^ii^	10 (10)
C412—C41—C411—N422^ii^	33 (10)	C421^ii^—C43—C432—N421^ii^	142 (22)
C431^ii^—C41—C411—N422^ii^	33 (11)	C41^ii^—C43—C432—C42^ii^	113 (3)
C422^ii^—C41—C411—N422^ii^	172 (22)	C431—C43—C432—C42^ii^	−151.0 (16)
C43^ii^—C41—C411—N411	−157 (12)	C4—C43—C432—C42^ii^	27.5 (17)
C4—C41—C411—N411	117 (13)	C412^ii^—C43—C432—C42^ii^	−159.9 (12)
C42^ii^—C41—C411—N411	90 (14)	C421^ii^—C43—C432—C42^ii^	−28 (14)
C412—C41—C411—N411	−68 (14)	C421^ii^—C432—N432—N421^ii^	166 (16)
C431^ii^—C41—C411—N411	−68 (14)	C42^ii^—C432—N432—N421^ii^	−176 (7)
C422^ii^—C41—C411—N411	71 (17)	N421^ii^—C432—N432—C421^ii^	−166 (16)
C43^ii^—C41—C411—C42^ii^	113 (3)	C42^ii^—C432—N432—C421^ii^	18 (10)
C4—C41—C411—C42^ii^	27.0 (16)		

Symmetry codes: (i) −*x*+1, −*y*+1, −*z*+1; (ii) −*x*+1, −*y*+2, −*z*.

### Hydrogen-bond geometry (Å, º)

**Table:**

*D*—H···*A*	*D*—H	H···*A*	*D*···*A*	*D*—H···*A*
C25—H25···N412	0.93	2.52	3.404 (16)	159
C26—H26···N421	0.93	2.31	3.23 (3)	170
C26—H26···N432^ii^	0.93	2.54	3.47 (3)	173
C36—H36···N412^iii^	0.93	2.48	3.352 (17)	157
C36—H36···N431^iv^	0.93	2.50	3.39 (2)	160

Symmetry codes: (ii) −*x*+1, −*y*+2, −*z*; (iii) *x*, *y*−1, *z*; (iv) −*x*+1, −*y*+1, −*z*.
